# Growth Disturbances Following Paediatric Anterior Cruciate Ligament Reconstruction: A Systematic Review

**DOI:** 10.7759/cureus.40455

**Published:** 2023-06-15

**Authors:** Vijay Patil, Praveen Rajan, Edward Hayter, Jonathan Bartlett, Sean Symons

**Affiliations:** 1 Trauma and Orthopaedics, Basildon University Hospital, Basildon, GBR; 2 Orthopaedic Surgery, University of Auckland, Auckland, NZL

**Keywords:** paediatric orthopaedics, knee surgery outcomes, paediatric knee, growth disturbance, anterior cruciate ligament (acl), anterior cruciate ligament reconstruction (aclr)

## Abstract

Growth disturbances after transphyseal paediatric anterior cruciate ligament (ACL) reconstruction have led to the development of physeal-sparing techniques. The aim of this study is to investigate growth disturbances following paediatric ACL reconstruction and identify associated risk factors.

A systematic search on PubMed, Scopus and Web of Science databases was conducted using Preferred Reporting Items for Systematic Reviews and Meta-Analyses (PRISMA) guidelines to identify case series reporting paediatric ACL reconstructions. Of 518 articles, 78 met the inclusion criteria, and data related to growth disturbances and graft failures were extracted.

A total of 2,693 paediatric ACL reconstructions resulted in 70 growth disturbances (2.6%): 17 were varus, 26 were valgus, 13 were shortening, 14 were lengthening and five patients had reduced tibial slope. Some patients showed deformities in more than one plane. Coronal plane deformities were seen more frequently with eccentric physeal arrest and lengthening with intraepiphyseal tunnelling. Shortening and reduced tibial slope were related to large central physeal arrest and anterior tibial physeal arrest, respectively. Sixty-two studies documented 166 graft failures in 2,120 reconstructions (7.8%). The extraphyseal technique was least likely to result in growth disturbances and graft failure.

Paediatric ACL reconstruction is a safe and effective treatment of rupture. Growth disturbances are least likely following extraphyseal tunnelling, and those resulting from transphyseal techniques can be minimised by reducing drill size, drilling steep and avoiding the physeal periphery. The insertion of hardware, synthetic material, or a bone plug through the drilled physis should be avoided. There is a greater need for robust long-term data collection, such as national ligament registries, to standardise practice and evaluate the risk of growth disturbance and re-ruptures in this treatment.

## Introduction and background

Early anterior cruciate ligament (ACL) reconstruction in the unstable paediatric knee reduces the risk of further instability and secondary meniscal and articular cartilage injury [1]. However, conventional ACL reconstruction using transphyseal drilling risks physeal injury with potential growth disturbance. This has led to the development of physeal-sparing intraepiphyseal and extraphyseal reconstruction techniques [2].

Literature evaluating growth disturbances following different methods of surgical ACL reconstruction in the skeletally immature is disproportionally influenced by selected case reports [3] and small case series [4]. It remains unclear as to what extent these growth disturbances are attributable to physeal insult with transphyseal tunnelling and how beneficial physeal-sparing techniques are in reducing growth disturbance or graft failure rates.

Primarily, the aim of this review is to assess the incidence, patterns and severity of growth disturbances at the tibial and femoral physes following ACL reconstruction in skeletally immature patients for different surgical techniques. Secondarily, we evaluated graft failure rates and risk factors in the same population.

## Review

Methods

A systematic review of PubMed, Scopus and Web of Science databases was performed using Preferred Reporting Items for Systematic Reviews and Meta-Analyses (PRISMA) guidelines. Abstracts including the terms “anterior cruciate ligament” or “ACL” and “immature” or “physes” or “physis” or “paediatric” or “pediatric” and “reconstruction” were collated. Articles assessing the growth disturbance following ACL reconstruction in patients with open physes were included.

**Figure 1:**
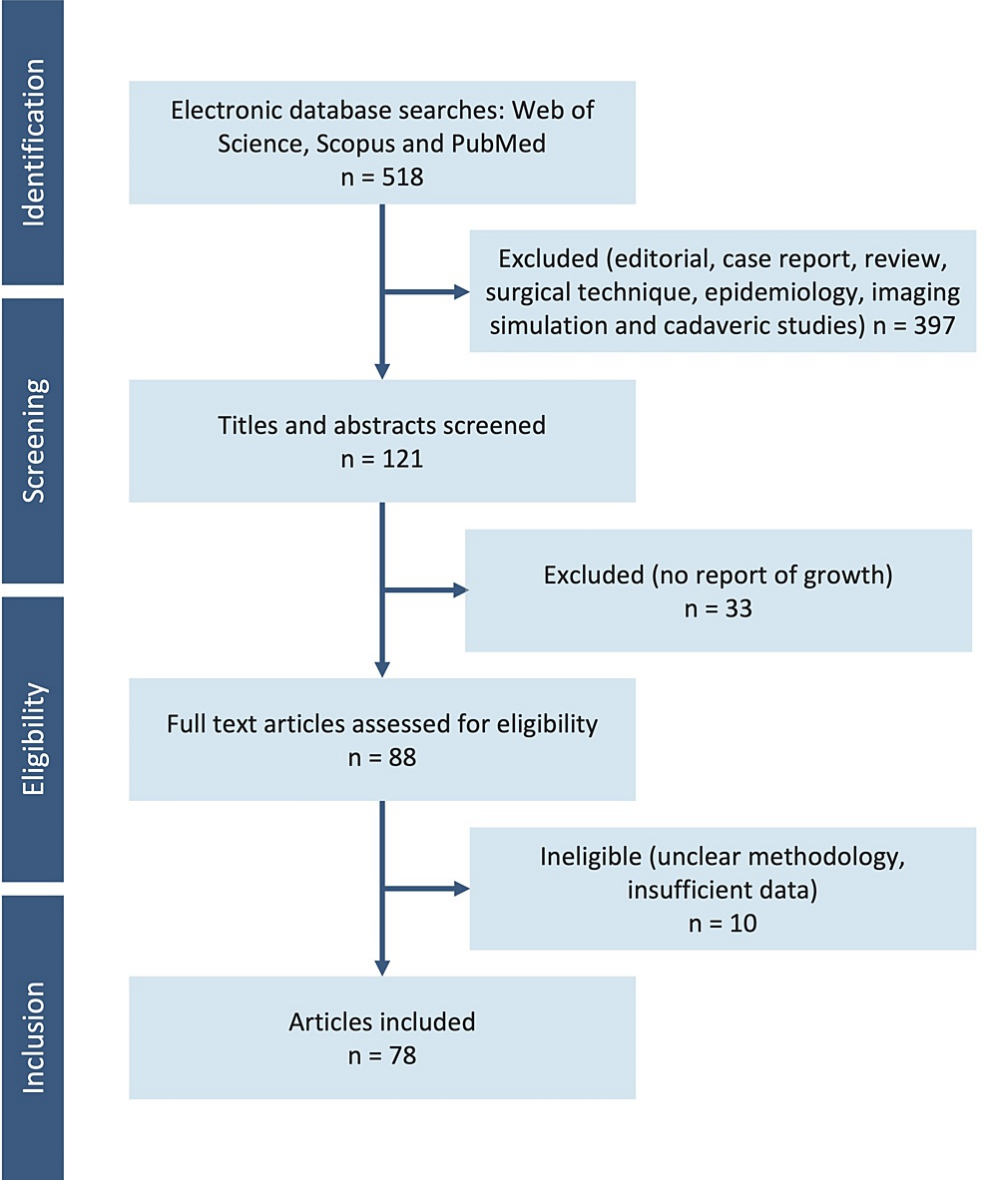
Study selection flow diagram according to the PRISMA statement PRISMA: Preferred Reporting Items for Systematic Reviews and Meta-Analyses

Two authors independently assessed studies for inclusion and extracted data. All languages were included without date restrictions, and the articles’ titles and abstracts were examined for relevance. Articles assessing growth disturbance following ACL reconstruction in patients with open physes were included (Figure 1). Additional relevant papers identified from the reference list of included articles were assessed for inclusion. This search initially yielded 518 articles published between 1994 and 2022. Following evaluation, 78 articles were included, and their texts were assessed [2,4-80].

Data on the number of procedures, mean age, sex and graft type were collected. The surgical technique utilised at both tibial and femoral ends was categorised into transphyseal, intraepiphyseal or extraphyseal. Cases of more than one procedure type were treated as separate datasets.

Growth disturbance was defined as either a 10 mm of leg length discrepancy, 3° difference in coronal plane angulation between the limbs or 3° difference in the posterior tibial slope between the limbs. Growth disturbances were divided into tibial and femoral growth disturbances. Data on graft rupture were collected when available.

Due to significant variations in reporting methods, surgical techniques, follow-up and outcome assessments, a descriptive analysis was performed.

Results

Our literature review identified 2,693 cases of ACL reconstruction (68.3% male) (mean age: 12.8 years) in 78 studies (67 retrospective and 11 prospective case series). The total number of ACL reconstructions that resulted in growth disturbances in our review was 70 (2.6%), of which 75.9% were male and the mean age was 12.1 years (Table 1) [4-26].

**Table 1:** Characteristics of reported growth disturbances by study GD, growth disturbance; M, male; F, female; mMPTA, mechanical medial proximal tibial angle; mLDFA, mechanical lateral distal femoral angle; MAD, mechanical axis deviation

Study (N=23)	Growth disturbance rate (GD/study total, %GD)	Tibial route	Femoral route	Graft type	Age, years	Sex	Growth disturbance (as reported in the study)
Bayomy et al. [5]	18/59, 30.5%	Transphyseal	Transphyseal	Hamstring autograft	12.5	M	12.6° loss of tibial slope
					13.6	M	5.9° loss of tibial slope
					14.8	M	9.9° loss of tibial slope
					13.7	M	5.4° loss of tibial slope
					14.3	M	6.1° loss of tibial slope
					11.3	M	5.6° varus mMPTA
					13.5	M	5.5° varus mMPTA
					8.8	M	10.9° valgus mLDFA and 5.9° varus mMPTA
					9.0	M	5.1° varus mMPTA
					8.3	F	6.4° valgus mLDFA
					Unstated	Unstated	5 patients: 5 mLDFA (3-5°)
					Unstated	Unstated	3 patients: 3 mMPTA (3-5°)
Roberti di Sarsina et al. [6]	1/20, 5%	Intraepiphyseal	Extraphyseal	Hamstring autograft	8.8	F	4° varus
Kumar et al. [7]	1/32, 31.5%	Transphyseal	Transphyseal	Hamstring autograft	Unstated	Unstated	6.2° valgus mLDFA
Mauch et al. [8]	1/49, 2%	Transphyseal	Transphyseal	Quadriceps tendon bone autograft	10.5	F	Valgus and flexed distal femur
Koch et al. [9]	2/13, 7.7%	Intraepiphyseal	Intraepiphyseal	Hamstring autograft	12.0	M	21 mm overgrowth and 6° varus
					Unstated	M	16 mm overgrowth
Sadykov et al. [4]	6/12, 50%	Transphyseal	Transphyseal	Patella bone tendon bone autograft	Unstated	Unstated	5 patients: 5 early growth arrest, 3 varus
				Synthetic graft	Unstated	Unstated	Early growth arrest and varus
Kohl et al. [10]	2/15, 13.3%	Transphyseal	Transphyseal	Quadriceps autograft	10.0	M	18 mm shortening and 6° valgus femur
					13.0	M	20 mm shortening
Liddle et al. [11]	1/17, 5.9%	Transphyseal	Transphyseal	Hamstring autograft	10.0	M	5° valgus
Wall et al. [12]	4/27, 14.8%	Intraepiphyseal	Intraepiphyseal	Hamstring autograft	9.4	M	27 mm overgrowth
					10.0	F	20 mm tibial overgrowth
					14.5	F	20 mm overgrowth
					11.7	M	Varus
Chambers et al. [13]	4/24, 16.7%	Transphyseal	Intraepiphyseal	Hamstring autograft	13.3	M	11.9 mm shortening
					12.2	M	12.2 mm shortening and 12.1 mm varus MAD
					10.1	M	18.1 mm shortening and 12.6 mm valgus MAD
					10.3	M	12.7 mm valgus MAD
Willson et al. [14]	2/23, 8.7%	Transphyseal	Intraepiphyseal	Hamstring autograft	10.0	M	22 mm overgrowth
					14.0	F	12 mm overgrowth
Cordasco et al. [15]	2/23, 8.7%	Intraepiphyseal	Intraepiphyseal	Hamstring autograft	Unstated	Unstated	16 mm femoral overgrowth
					Unstated	Unstated	18 mm femoral overgrowth
Pennock et al. [16]	1/26, 3.8%	Intraepiphyseal	Intraepiphyseal	Hamstring autograft	Unstated	Unstated	12 mm overgrowth
Dei Giudici et al. [17]	1/19, 5.3%	Transphyseal	Transphyseal	Hamstring autograft	Unstated	Unstated	15 mm overgrowth
Holwein et al. [18]	3/37, 8.1%	Transphyseal	Transphyseal	Hamstring autograft	Unstated	Unstated	3 patients: 2 valgus, 1 varus
Volpi et al. [19]	3/71, 4.2%	Intraepiphyseal	Transphyseal	Hamstring autograft	Unstated	Unstated	3 patients: 3 valgus (>2°)
Andrews et al. [20]	2/8, 25%	Transphyseal	Extraphyseal	Iliotibial band and Achilles allograft	13.3 (mean)	Unstated	2 patients: 10 mm femoral overgrowth, 12 mm femoral shortening
Lemaitre et al. [21]	2/14, 14.3%	Transphyseal	Transphyseal	Hamstring autograft	13.3	M	Valgus
					11.5	F	Valgus
McIntosh et al. [22]	1/16, 8.7%	Transphyseal	Transphyseal	Hamstring autograft	Unstated	Unstated	15 mm overgrowth
Saad et al. [23]	2/19, 10.5%	Intraepiphyseal	Intraepiphyseal	Hamstring autograft	Unstated	Unstated	2 patients: 1 mm overgrowth, 6° varus
Sasaki et al. [24]	8/102, 7.8%	Intraepiphyseal	Intraepiphyseal	Hamstring autograft	11.0	F	8° varus
					13.0	M	5° valgus
					14.0	M	4° valgus
					14.0	M	6° varus
		Transphyseal	Transphyseal		12.0	F	4° varus
					15.0	F	5° valgus
					15.0	F	4° valgus
					14.0	M	5° valgus
Lanzetti et al. [25]	2/42, 4.8%	Intraepiphyseal	Extraphyseal	Hamstring autograft	Unstated	Unstated	2 patients: 3° valgus, 4° valgus
Wilson et al. [26]	1/57, 1.7%	Transphyseal	Extraphyseal	Iliotibial band and hamstring autograft	Unstated	Unstated	Shortening and valgus

**Figure 2:**
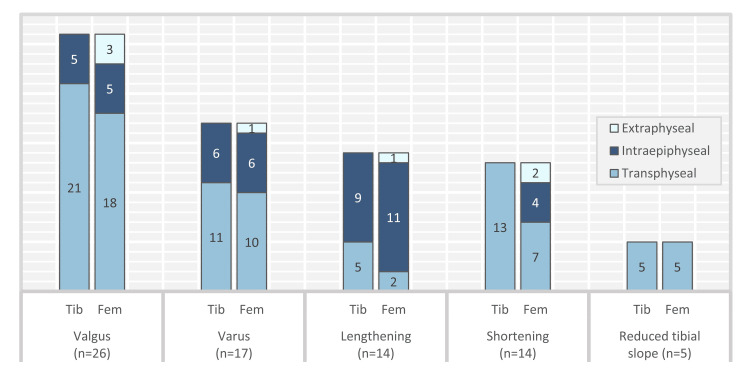
Growth deformity by tibial and femoral physeal procedure Tib, tibial physeal route; Fem, femoral physeal route

Valgus deformity was the most common growth disturbance (Figure 2). Femoral Intraepiphyseal tunnelling was associated with higher rates of growth disturbance compared to other techniques (Table 2). The combination of tibial transphyseal with femoral intraepiphyseal graft routing had the highest rates of growth disturbances (Table 3). Extraphyseal routing of the graft in both tibial and femoral ends showed minimum resultant disturbances. Sixty-two studies reported 166 graft ruptures from 2,120 cases with an overall risk of 7.8%. This risk was highest with transphyseal graft routing and least with extraphyseal techniques (Table 4). Hamstring autograft was the most used graft but also showed higher rates of rupture compared to the other grafts (Table 5).

**Table 2:** Growth disturbance rate by physeal procedure

Physeal route	Procedures	Mean age (years)	Growth disturbance	Rate of growth disturbance (%)
Tibial transphyseal	1,765	12.8	48	2.7%
Tibial intraepiphyseal	846	11.8	22	2.6%
Tibial extraphyseal	82	10.7	0	0%
Femoral transphyseal	1,508	13.1	43	2.9%
Femoral intraepiphyseal	632	11.8	21	3.3%
Femoral extraphyseal	553	10.7	6	1.1%
All	2,693	12.8	70	2.6%

**Table 3:** Growth disturbance rate by physeal procedure combination

Tibial route	Femoral route	Procedures	Growth disturbance	Rate of growth disturbance (%)
Transphyseal	Transphyseal	1,359	40	2.9%
Transphyseal	Intraepiphyseal	246	9	3.7%
Transphyseal	Extraphyseal	160	3	1.9%
Intraepiphyseal	Transphyseal	80	0	0%
Intraepiphyseal	Intraepiphyseal	455	15	3.3%
Intraepiphyseal	Extraphyseal	311	3	1%
Extraphyseal	Extraphyseal	82	0	0%

**Table 4:** Graft rupture rate by physeal procedure

Physeal route	Procedures with reported graft rupture rates	Graft rupture	Rate of graft rupture (%)
Tibia transphyseal	1,270	111	8.7%
Tibia intraepiphyseal	771	51	6.6%
Tibia extraphyseal	79	4	5.1%
Femoral transphyseal	1,024	95	9.3%
Femoral intraepiphyseal	596	41	6.9%
Femoral extraphyseal	500	30	6%
All	2,120	166	7.8%

**Table 5:** Graft rupture rate by graft type

Graft type	Procedures with reported graft rupture rates	Graft rupture	Rate of graft rupture (%)
Hamstring autograft	1,399	130	9.3%
Iliotibial band autograft	311	20	6.4%
Iliotibial band and hamstring autograft	57	3	5.3%
Patella bone tendon bone autograft	76	4	5.3%
Fascia lata autograft	20	1	5%
Patella autograft	78	3	3.9%
Quadriceps tendon autograft	146	5	3.4%
Achilles allograft	19	0	0%
Patella allograft	10	0	0%
Hamstring autograft and synthetic augment	2	0	0%
Quadriceps tendon autograft and synthetic augment	2	0	0%
All	2,120	166	7.8%

Discussion

This is the largest systematic study analysing paediatric ACL reconstruction with regard to growth disturbance and graft rupture. We used the three medical databases that provided us with the most accurate, up-to-date and broadest coverage [81].

Alterations in the growth around the physis can be due to three mechanisms: arrest, boost or deceleration [82]. Growth arrest due to localised physeal bridge formation may cause growth discrepancy throughout the remaining skeletal maturation. Localised growth arrest closer to the periphery of the physis may result in angular deformity and length discrepancies if affecting the centre. Local physeal hypervascularization after trauma or surgery may stimulate increased growth and limb length and becomes apparent two years following the insult. This leads mainly to a leg length discrepancy [22] but can also lead to angular deformities, particularly tibial valgus deformity [83,84].

Undergrowth due to the tenodesis effect from graft tension across the open physis has been reported [85,86], whereby shortening and femoral valgus and tibial varus deformities can develop in the absence of physeal arrest. Based on a questionnaire survey of 140 surgeons, Kocher et al. [87] proposed the causes of growth disturbances as fixation hardware or bone plugs across the physis, large tunnels and lateral extra-articular tenodesis. Moreover, there may be an additional influence of the Hueter-Volkmann effect [88], with local physeal distraction stimulating growth and compression suppressing it, thus resulting in angular growth disturbances.

A limb length discrepancy of 20 mm is described as clinically significant in adults due to its effect on gait, lower back pain and hip and knee arthritis [89,90]. However, as growth discrepancy may continue to increase until skeletal maturity beyond the remit of most studies, we defined growth disturbance at a lower threshold (10 mm length discrepancy and 3° angular difference), in line with current literature [91,92].

Accurate skeletal age assessment at the time of surgery may have an important bearing on its effect on the effective growth disturbance. However, there was significant inhomogeneity in reporting skeletal age in the studies, making it difficult to interpret its relationship with the occurrence of growth disturbances. A recent description of peak height velocity (PHV) of pubertal growth has attracted attention in recent years for growth disturbances. During this period, the child experiences the fastest upward growth in their stature characterized by a short and rapid acceleration, followed by a more gradual deceleration, and it has been described to predict final height as 90% of the height at the initiation of the growth spurt [93]. Knapik et al. [94] have recently shown that skeletal age assessment using a combination of chronologic age, sex and central peak value (CPV) using three major undulations on plain knee radiographs provided a more accurate estimation of 90% of final height when compared with the Greulich and Pyle method.

Coronal plane angular deformities, particularly valgus, were found to be more common than length disturbance in our study. Severe valgus deformities have been reported with transphyseal bone plugs and transfixing screws [95]. Angular deformity with broad physeal arrest due to the proximity of the drilled area to the physis [3] in an intraepiphyseal femoral tunnel with resulting valgus and transphyseal bone plug across the tibial tunnel resulting in varus deformity has been documented [96].

Only one study [5] reported sagittal plane deformity of reduced tibial slope in five out of 59 cases using transphyseal techniques at both ends. Shifflett et al. [27] noted similar genu recurvatum in two reports of transphyseal tibial biointerference screws resulting in anterior tibial physeal growth arrest. Early closure of tibial physis was also shown by Kocher et al. [87] in a survey with a transphyseal staple across the apophysis or suturing to the tibial periosteum.

We found that angular deformities were consistent with asymmetric physeal growth disturbance. Valgus, varus and reduced anterior tibial slope appeared to be attributed to the physeal arrests in the lateral distal femur, medial proximal tibia and anterior proximal tibia, respectively. In cases of significant lengthening deformities, intraepiphyseal tunnelling was used in 77% of femurs and 64% of tibia. In cases with significant shortening deformities, all tibial tunnels and 54% of femoral tunnelling were transphyseal. Central physeal disturbances are likely to be attributable to such non-angular growth disturbances.

Reducing the quantitative physeal insult by keeping the transphyseal tunnel size to less than 6 mm was demonstrated by Lo et al. [97] and Guzzanti et al. [28], resulting in no significant growth disturbance. Leaving a transphyseal tunnel empty was noted to result in greater shortening and valgus angulation when compared to filling the defect with soft tissue grafts [98,99] in two animal experimental studies.

Greater volumetric femoral physeal damage has been shown to be related to angular deformities [27]. Abebe et al. [100] described oblique drilling as causing more physeal destruction, via the anteromedial portal, compared with the vertical transtibial technique.

Shea et al. [101] postulated that tibial drill holes that started more medial and distal with a steeper angle of inclination reduced the volumetric physeal damage and crossed the physis more centrally, away from the vulnerable periphery of the physis.

Bone plugs and hardware crossing the physis have been strongly associated with localised growth arrest, resulting in significant growth disturbance frequently requiring corrective procedures [4,87,95,96].

The role of tenoepiphyseodesis by transphyseal graft was demonstrated in animal studies [85,86]: graft tension of 80 N caused growth disturbance, whereas no growth disturbances were seen in ruptured reconstructions. Seil et al. [98] demonstrated no growth disturbance in an ovine study if intraoperative ACL tension was kept at 40 N.

Role of Tunnelling Techniques

Bony bridge formation has been reported in transphyseal techniques [29,30] but is shown to resolve spontaneously due to the high expansion forces acting across the physis in preadolescents [102].

Growth deformities resulting from transphyseal ACL reconstruction can be minimised by following these principles: minimise volumetric damage to the physis; keep drill size under 6 mm and drill angle steep; avoid drilling the physeal periphery; avoid inserting a bone plug, hardware or synthetic material through the drilled physis; and do not leave the drill hole empty.

During intraepiphyseal drilling, an optimum gap should be maintained between the drilled bone and physis to avoid the effects of hypervascularisation or bony bridge formation [3]. Excess tensioning across physis with the intraepiphyseal graft placement may be associated with growth acceleration.

Extraphyseal techniques are shown to have minimum growth disturbances; however, excessive graft tension should be avoided to negate the tenodesis effect.

Graft Types

Although hamstring autograft is the most commonly used graft in our review, quadriceps and patellar tendon grafts without a bone block remain a valid option. If a bone block is employed, placement of the block through the growth plate must be avoided to prevent early growth plate fusion [103]. The use of an iliotibial band in the extraphyseal technique has shown satisfactory outcomes [31], but it requires large skin incisions. The International Olympic Committee (IOC) recommended against the use of cadaver allografts in immature children, which have been attributed to poor clinical outcomes [104]. Moreover, synthetic grafts have a high risk of growth arrest and should be discouraged in pediatric ACL reconstruction [103]. Living-donor hamstring tendon allografts may have advances over their cadaver counterparts but raise ethical questions, and long-term outcomes need further assessment [105,106]. The preservation of the distal tibial insertion of hamstrings has shown promising results in adults [107], and this technique minimises proximal tibial physis insult. However, there is not enough literature support to recommend this technique in the paediatric population.

Re-rupture

We found that the overall risk of paediatric graft re-rupture was 7.8% in our review. However, due to limited follow-up, the true failure rate is likely to be higher over longer follow-ups. Kaeding et al. [108] reported an 8.5% re-rupture rate from 140 patients in five studies, in which there was no significant difference noted between physeal-sparing and transphyseal techniques. We, however, noted significantly higher graft failures in transphyseal techniques compared to physeal-sparing ones. In our review, no graft re-rupture was noted when 29 allografts were used for reconstructions. This contrasts with a recent systematic review that demonstrated significantly higher odds of graft rupture with allografts when compared with hamstrings or bone-patellar tendon-bone (BTB) autografts [109].

Limitations

Due to inconsistent duration of follow-up and lack of follow-up till skeletal maturity, evaluation of the relationship between growth disturbance and patient age at the time of procedure could not be performed in this review. The issue is further compounded by variable methods used to assess growth disturbances, the most common method being clinical and plain radiological examinations. This may be sufficient to identify clinically significant leg length discrepancies; however, the subtle angular deformity may have been missed with possible underreporting of growth deformities in our cohort, particularly given that angular deformities were more common than leg length discrepancies. Our search identified only retrospective and prospective case series, limiting the level of evidence reviewed in this study. We included articles that reported growth disturbances; due to publication bias, there is a risk of under- or overestimating the rates of growth disturbances and graft failure, limiting the generalisability of results.

## Conclusions

Anterior cruciate ligament reconstruction is recommended in skeletally immature patients with unstable ACL injuries to prevent secondary damage. However, growth disturbance due to physeal insult remains a known complication, the incidences of which are relatively lower in extraphyseal techniques. This study highlights the need for further studies involving thorough clinical and radiographic assessment of these patients following surgical intervention, with follow-up to skeletal maturity. There is a greater need for the establishment of national paediatric ligament registries to standardise pre-operative and post-operative assessment and further characterise the risks of growth disturbance and re-rupture.
